# CT Findings of Patients Treated with Irreversible Electroporation for Locally Advanced Pancreatic Cancer

**DOI:** 10.1155/2015/680319

**Published:** 2015-11-05

**Authors:** Olaguoke Akinwande, Shakeeb S. Ahmad, Tracy Van Meter, Brittany Schulz, Robert C. G. Martin

**Affiliations:** ^1^Division of Interventional Radiology, Department of Radiology, University of Pittsburgh Medical Center, Pittsburgh, PA 15213, USA; ^2^Division of Interventional Radiology, Department of Radiology, Johns Hopkins University, Baltimore, MD 21287, USA; ^3^Department of Radiology, University of Louisville School of Medicine, Louisville, KY 40202, USA; ^4^Division of Surgical Oncology, Department of Surgery, University of Louisville School of Medicine, Louisville, KY 40202, USA

## Abstract

*Introduction*. In patients with locally advanced pancreatic cancer (LAPC), IRE has been shown to be safe for local disease control and palliation. As IRE continues to gain acceptance it is important to characterize the expected imaging findings. *Materials and Methods*. A review of our prospective soft tissue ablation registry from July 2010 to June 2013 was performed on patients who had undergone IRE for LAPC. Five masses treated with intraoperative IRE ablation for pancreatic tumors that underwent CT imaging before and after ablation were reviewed. *Results and Discussion*. Following IRE, the postablation bed is larger than the original ablated tumor. This ablation zone may get smaller in size (due to decreased edema and hyperemia) in the following months and more importantly remains stable provided there is no recurrence. In cases of recurrent disease there is increased size of the ablation bed, mass effect, and new or worsening vascular encasement or occlusion. *Conclusion*. CT imaging remains the best current imaging modality to assess post-IRE ablation changes. Serial imaging over at least 2–6 months must be employed to detect recurrence by comparing with prior studies in conjunction with clinical and serum studies. Larger imaging studies are underway to evaluate a more ideal imaging modality for this unique patient population.

## 1. Introduction

Pancreatic cancer carries a challenging prognosis since it is most often diagnosed at an advanced stage of disease [1, 2]. At the time of diagnosis, 35% of patients have locally advanced pancreatic cancer (LAPC)/stage III disease with abutment or encasement of the celiac axis and/or the superior mesenteric artery (SMA) and/or unreconstructable portal venous encasement [3, 4]. This encasement limits patients surgical or consolidative options [5].

Recent options for attempts at enhanced local control have been external beam radiation therapy, High Frequency Ultrasound, and ethanol injections. Recently thermal based ablative techniques, radiofrequency ablation (RFA), and microwave have been used but have been found to induce a high incidence of injury to adjacent vital structures: blood vessels and nerves [6–11]. As a result, these techniques have not been shown to provide any benefit and are limited in their effectiveness for palliation [7, 8].

Irreversible electroporation (IRE) is a nonthermal technique which utilizes electrical pulses to destroy cells by causing membrane permeability ultimately leading to apoptosis. IRE has been shown to have good efficacy and safety in the management of patients with LAPC [12]. Furthermore, the potential for improved survival rates has been reported in patients treated with IRE ablation combined with chemotherapy and/or chemoradiation therapy compared with patients treated with chemotherapy or chemoradiation therapy without IRE ablation [13]. These promising findings have led to increased utilization of IRE both intraoperatively and percutaneously [14, 15]. Since IRE is relatively new to the field of locoregional therapy, post-IRE imaging findings are scant [16, 17]. The purpose of this study is to characterize the expected Computed Tomography (CT) imaging findings in the surveillance of patients with locally advanced pancreatic cancer (LAPC) treated with irreversible electroporation (IRE).

## 2. Materials and Methods

### 2.1. Patients

A review of our prospectively maintained soft tissue ablation registry from May 2010 to May 2013 was performed. Institutional Review Board approval and patient informed consent were obtained. There were a total of 253 patients treated during that time interval. Five representative patients with LAPC treated with IRE in situ (IRE ablation without surgical resection for adenocarcinoma) were selected for this interim report. The CT imaging for these patients includes an immediate pre-IRE scan (within 7 days of procedure), a discharge CT scan (approximately 7–10 days after procedure) then serial CT scans every 3 months for the next 2 years with serum CA19-9. If the CT scan was equivocal or concerning for recurrence, a CT-PET scan was obtained for confirmation of the patient's current disease status. The selection of patients for this study was based on the availability of at least one preprocedural CT image and at least one or several surveillance CT images. This was necessary because, to discriminate between the target lesion and the initial postablation bed, preoperative images and immediate postoperative images (usually done within 1 month) were needed. By the same token, to compare changes in the ablation zone, the immediate postoperative scan and at least one-follow-up CT scan were needed. All patients had locally advanced pancreatic adenocarcinoma involving the pancreatic head.

### 2.2. IRE Ablation Procedure

IRE was performed on patients with LAPC. A three-phase CT scan was performed less than 7 days before IRE and after induction chemotherapy to ensure the persistence of locally advanced pancreatic cancer and to rule out macroscopic metastatic disease that may have ensued in the interim (pancreatic cancer typically does not respond to induction chemotherapy). Also, patients with masses greater than 3.5 cm were excluded as candidates for therapy based on our institutional protocol [18]. Intraoperative IRE was performed by open laparotomy through a superior midline incision. High definition intraoperative ultrasound was utilized to reassess tumor size and to confirm that the pancreatic tumor in question truly meets the criteria for LAPC. All patients underwent intraoperative IRE ablation without surgical resection. Our criterion for decision making was previously published by Martin [18]. With ultrasound guidance, 4 or 5 needles are introduced and spaced approximately 2 cm (±0.3 cm) apart, bracketing the tumor with the adjacent vital structures. A 0.5 cm to 1 cm margin is included in the IRE field. IRE is performed with the Nanoknife system (AngioDynamics, Lanthan) utilizing 1,500 volts/cm for a delivery of >90 pulses (pulse width 70–90 ms). A repeat ultrasound with Power Doppler is performed to confirm preserved flow and patency to the vital structures.

### 2.3. Imaging Methods

We decided to follow our patients with CT scan because there is better availability and cost benefit compared to PET-CT and MRI. Standard workup for our patients includes a three-phase CT scan with pancreatic protocol at the time of diagnosis, which allows us to both diagnose and stage the patients appropriately. For most patients three-phase CT scan was also obtained in the immediate postoperative period (less than 1 month postoperatively) to assess the patency of vital structures and to establish a baseline of the postablation bed. No true ablation efficacy can be confirmed at this immediate CT scan since ongoing electroporation effects (most commonly cellular apoptosis) are seen in this immediate phase. Subsequent surveillance studies were scheduled at 3-month intervals using the same protocol. All CT images were obtained with a helical scanner (Somatom Sensation 64, Siemens) before and after a bolus injection of 100 mL of nonionic contrast (ioversol [Optiray 350]; Mallinckrodt Inc., Hazelwood, Missouri) intravenously at a rate of 3 mL/sec. After contrast injection, two spiral CT scans were obtained during the arterial phase and portal venous phase at 30 and 70 seconds, respectively, after the initiation of the injection. Precontrast and arterial phase acquisitions were performed on the upper abdomen to include the pancreas and liver; portal venous phase imaging included the abdomen and pelvis. Contiguously reconstructed sections were obtained through the pancreas (5 mm × 5 mm for noncontrast and 2 mm × 2 mm for arterial and venous); coronal reconstructions for each phase were performed. Each spiral acquisition was accomplished during a breath hold. We do not believe in cross comparing CT scan to MRI or CT scan to potential PET scan unless there are abnormal signs of recurrence; most of these patients were followed with serial triphasic CT scan alone and were not comprehensively evaluated with both CT and MRI. Given that a majority of these patients did not have preoperative MRI most commonly because of the metal biliary stents in place, we have found that triphasic CT scan is the most consistent, most reliable way to image this unique subset of patients who were treated with IRE.

### 2.4. Image Analysis

Pre-IRE and post-IRE CT studies were reviewed by three radiologists (TVM, BS, and OA). These physicians were not involved with the ablation process and were blinded to the final clinical assessment and serum tests at the time of interpretation. Therefore, for the sake of description, we will describe the treated lesion in the immediate postablation scan as the “ablation bed”; the same area in subsequent surveillance studies will be termed the “ablation zone.” Post-IRE imaging of the pancreas has been reported to be quite challenging due to the acute inflammatory changes seen from the first through tenth postoperative day. Further, an ongoing apoptotic process that persists for up to 6–8 weeks after electroporation has been reported [19, 20].

The lesions we measured had poorly defined margins, which made measurement cumbersome and may affect the accuracy and reproducibility of the measurement. Therefore, we utilized previously published criteria for our evaluation. Ablation success was defined as the ability to deliver the intraoperative therapy and within 3 months to have no evidence of macroscopic tumor by CT imaging. According to previously reported data, local recurrence after ablation most often presents as new tissue in the periphery of the treated lesion and/or apparent enlargement of the ablation defect, in other words a change in size or morphology from a previously “stable” ablation zone [21–23]. Those imaging criteria along with a rising CA 19-9 value and the clinical presentation (return of back pain, jaundice, or worsening gastric emptying) were used to determine incomplete electroporation or local recurrence.

## 3. Results and Discussion

### 3.1. Results

Five patients underwent intraoperative IRE ablation for adenocarcinoma from May 2010 to May 2013. The arterial phase study provided the best images because it was easier to separate the relatively hypoattenuating ablation zone from the adjacent vasculature. Also, it provided the opportunity to scrutinize the adjacent vessels for any new or increasing stenosis.

**Figure 1:**
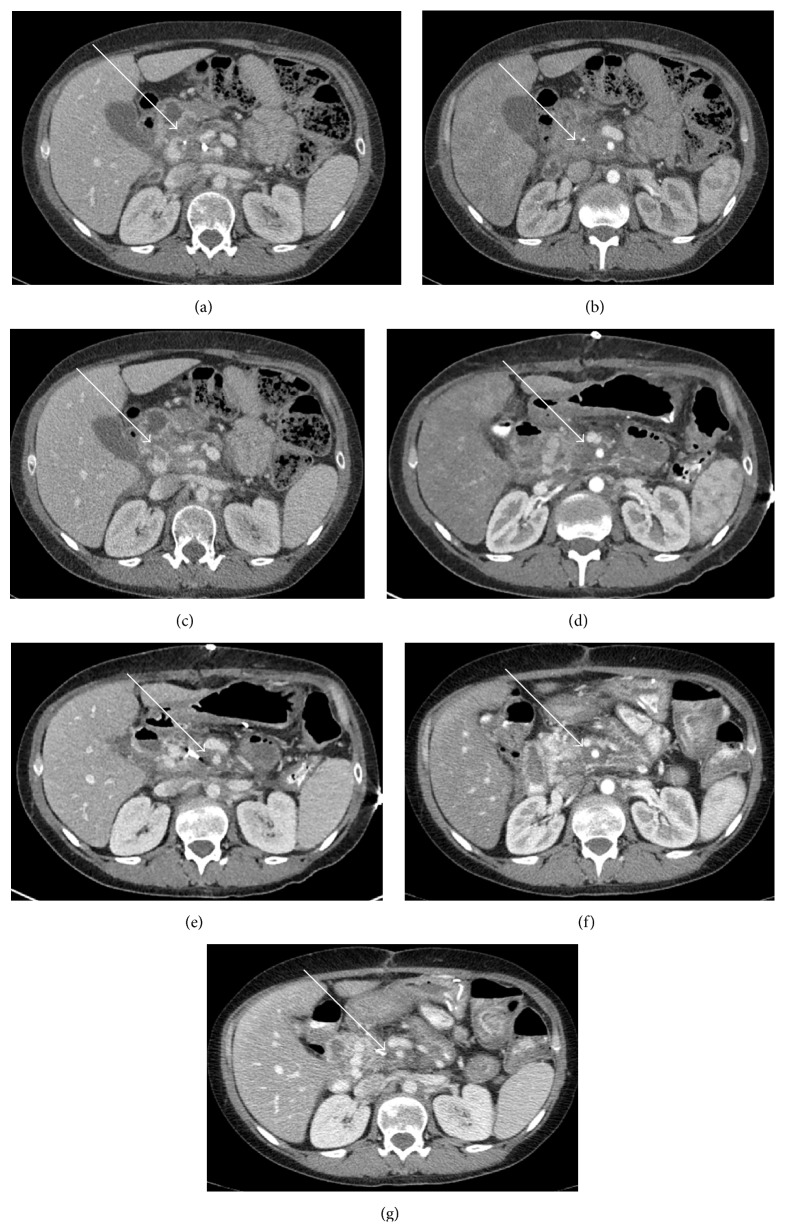
(a) Preoperative CT scan in the portal venous phase displaying abnormal soft tissue in the inferior pancreatic head (white arrow) with a nearby fiducial marker. (b) Arterial phase image displaying soft tissue encasement (white arrow) of superior mesenteric artery (SMA). Another preoperative scan (c) displaying soft tissue encasement along splenic vein and posterior to the portal confluence (white arrow). Immediate postoperative scans in arterial (d) and venous phases (e) displaying an increase in hazy soft tissue in the postablation bed (white arrows) and continued encirclement of SMA. A 2-month postoperative scan in arterial (f) and venous (g) phases displaying similar irregular, hazy, amorphous soft tissue stranding but with decreased size of the postablation zone (white arrow) consistent with no recurrence.

**Figure 2 fig2:**
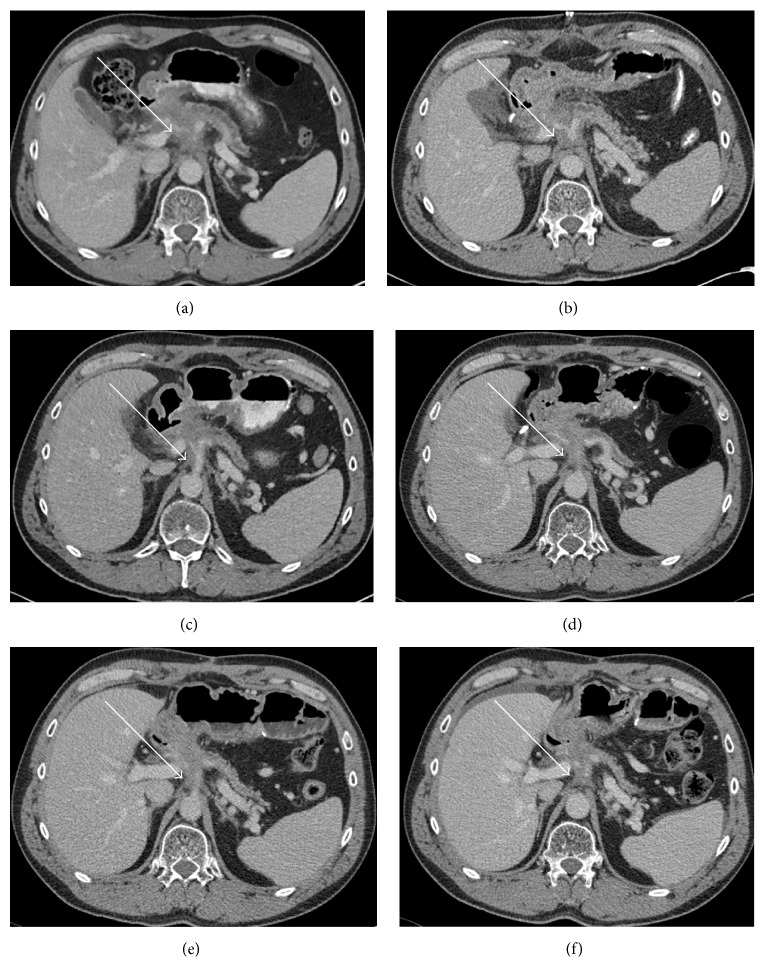
(a) Preoperative CT scan demonstrating soft tissue in pancreatic neck (white arrow) with distal pancreatic obstruction along with encasement of the celiac axis and its branches. (b) Immediate postoperative scan demonstrating irregular, hazy soft tissue with ill-defined margins in the postablative bed (white arrow). (c) Three-month follow-up scan showing similar hazy amorphous tissue in the postablation zone (white arrow) which has decreased in size. Further surveillance scans at five months (d), eight months (e), and ten months (f) demonstrating similar appearance of hazy, amorphous soft tissue which has remained stable in size (white arrows) with no signs of recurrence.

**Figure 3 fig3:**
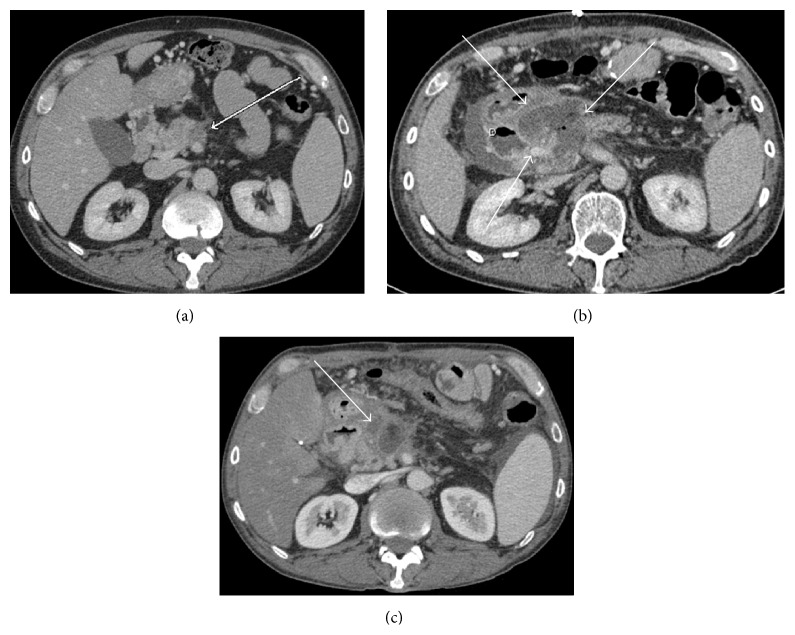
(a) Preoperative CT scan demonstrating soft tissue in the posterior aspect of the proximal pancreatic body (white arrow) extending posteriorly to surround at least 180 degrees of the proximal to midsuperior mesenteric artery. (b) Immediate postoperative CT scan demonstrating a large fluid collection in ablative bed with foci of internal gas. Ablative zone (white arrows) has been demarcated from nearby duodenum (D). (c) Three-month follow-up scan showing markedly decreased ablative zone with decreased size of the fluid collection (white arrow) consistent with no recurrent disease.

**Figure 4 fig4:**
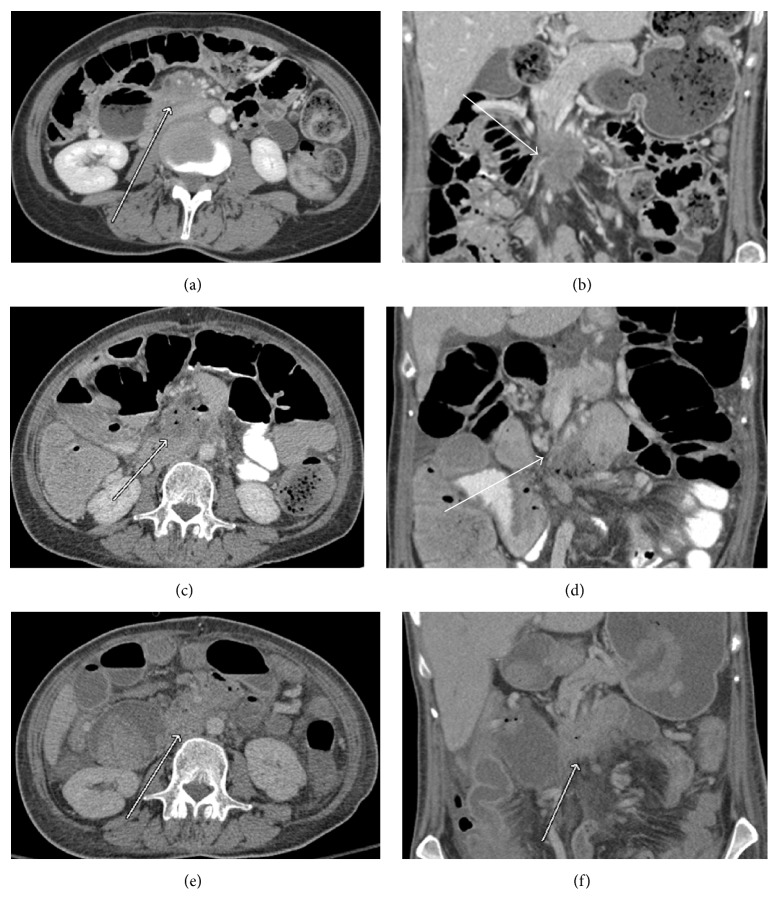
(a) and (b) Preoperative CT scans in axial and coronal planes showing soft tissue mass (white arrow) in the pancreatic uncinate process extending into the small bowel mesentery with vascular encasement. (c) and (d) Immediate postoperative CT scans demonstrating a slight fluid collection in postablative zone (white arrow) with ill-defined margins and internal gas compatible with liquefaction from ablation procedure. (e) and (f) Two-month follow-up scans demonstrating a smaller ablation zone (white arrow) with increased enhancement and less gas suggesting no recurrent disease.

**Figure 5:**
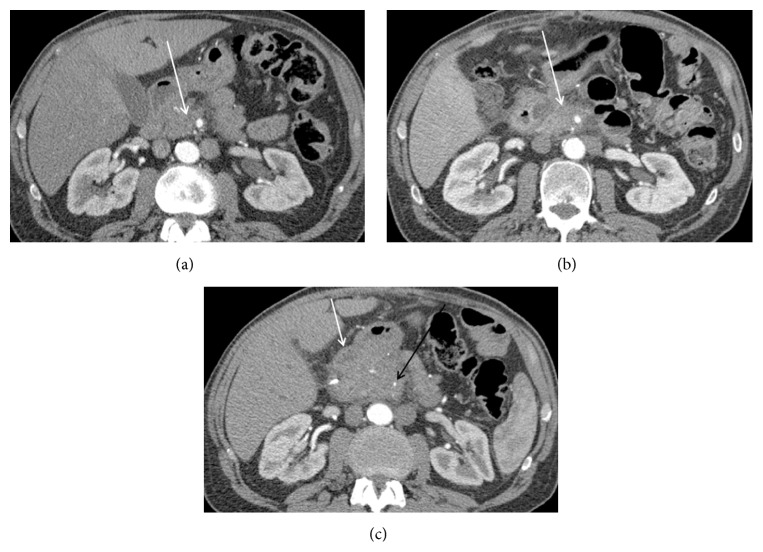
Features worrisome for recurrence. (a) Pre-op CT, a soft tissue mass (white arrow) is seen surrounding the SMA; (b) immediately post-op, hazy amorphous soft tissue (white arrow) is seen surrounding the SMA. There is some enhancement of the ablation bed; (c) 2 months post-op, there is new narrowing of the SMA (black arrow) with enlarging soft tissue at the postablation zone. Its anterior borders cannot be separated from adjacent bowel (white arrow). Increased volume and new narrowing of a vessel are suggestive of recurrence.

As Figures 1 [Fig fig2] [Fig fig3] [Fig fig4]–5 demonstrate, the immediate postablation bed and zone are invariably larger than the original ablated tumor. We remain descriptive because the entire bed was extremely difficult to measure owing to the amorphous, irregular nature of the ablation. Moreover, the ablated tissue is not within an encapsulated organ; therefore, the ablation zone does not have defined borders as seen after, for instance, liver ablation. Four patients that showed continued stable disease are highlighted in Figures 1 through 4. An amorphous, hypoattenuating region with irregular shape persisted in subsequent CT scans in all patients with “stable disease.” Moreover, the ablation zone was typically smaller (due to decreased edema, hyperemia, and granulation tissue) than the immediate postablation bed in the following months and remained stable provided there was no recurrence. Imaging findings demonstrating recurrence are shown in Figure 5. Along with persistent irregular shape the ablation zone showed increased tumor bulk and extension as well as new mass effect (new narrowing of a blood vessel). Enhancement of the ablative bed was variable and often showed increased enhancement in the three-month and longer follow-up images. This was felt to be related to development of granulation tissue and fibrosis.

### 3.2. Discussion

IRE has been gaining popularity in large part because of its nonthermal properties. As it is nonthermal, it is less susceptible to perfusion-mediated tissue cooling (heat sink) potentially allowing the ablative zone to be more predictable [24]. This permits the ability to ablate tumors close to vital organs. Locally advanced pancreatic cancer (LAPC) is a good candidate to demonstrate this novel ablative technique because surgery and thermal ablative techniques are challenging in the patient subset. Since ablative therapies for pancreatic carcinoma are becoming quite prevalent, it is important that radiologists are aware of the imaging findings so as to better direct the referring physician. Some papers have been published on imaging findings after post-IRE ablation of the liver [16, 17]; however, we cannot apply those principles to pancreatic cancer ablation. The main reason for this difference is the significant heterogeneity of the pancreatic tumor and tissue. Given the location, all stage pancreatic cancers are not surrounded by normal pancreatic parenchyma and the retroperitoneal tissue, surrounding vessels, and duodenum all create variability on imaging, which is further challenged by the electroporation effects.

In our study, we found that the postablation bed is larger in volume than the initial mass. This is expected because it contains the tumor and the ablative margin. The postablation bed and zone appear irregular, amorphous, and hazy without margins or true boundaries. The ablation zone may decrease in size from the initial post-op bed to the initial surveillance study as the surrounding edema/fluid and inflammation resolve revealing the true ablation zone; however, as mentioned above, since there have been reports of an ongoing apoptotic process that persists up to 6–8 weeks after ablation, it will not be unusual to see some increase in volume in surveillance [19, 20]. Therefore, size is considered secondary in the CT evaluation for this reason and because the postablative bed/zone has poorly defined margins, making objective imaging assessment (size, attenuation) cumbersome. This may undoubtedly affect the accuracy and reproducibility of the measurement. Nonetheless, any increase in volume after stabilization of the postablation zone is considered worrisome for recurrence (Figure 5).

Other clues that may suggest recurrence are any new encasement or narrowing of adjacent vessels or any subjective extension of soft tissue outside the boundaries of the previously established baseline ablation zone. However, in patients that have undergone prior radiation therapy or undergo post-IRE radiation therapy, persistent isolated narrowing (without other worrisome findings) is not always recurrence and must be followed with serial imaging, clinical evaluation, and CA19-9 serum tumor markers. Vessels within and adjacent to the ablation bed may show narrowing immediately after the procedure, but this should resolve or at least remain stable in subsequent studies. Often, if narrowing of a vessel is seen with the index tumor it will often persist after ablation. For equivocal cases, PET/CT may play a role in differentiating postablative changes from recurrence [25].

This study has several limitations. First is our small sample size; however, the ongoing study is the single largest evaluation of this population to date. Second, it was difficult to make an objective imaging assessment for the reasons detailed above. We believe that this pictorial interim report has value because it is the first paper on this topic and may provide a foundation for future studies. Further, as we gather more data at our institution, we will publish our results when our findings mature. Another limitation was that the efficacy of the ablative therapy relies only on imaging characteristics. Our imaging findings are related to the presence or absence of macroscopic disease and there is no way to prove by imaging whether or not small volume disease remains at the ablation bed. However, what we cannot see through imaging cannot be treated; therefore our goal is to provide a guide as to when the imaging characteristics are worrisome enough to intervene. Despite these limitations, we still believe that this is a worthy introduction to the understanding of postablative pancreatic lesions. These therapies are becoming prevalent and it is important that we understand the imaging characteristics. It is difficult to predict if these postablative changes will apply to other ablative technologies such as RFA or microwave; therefore it will be interesting to compare our findings with subsequent studies or case series. Comparing our findings with that of a percutaneous approach is also of interest.

## 4. Conclusion

We conclude that CT imaging may be a useful tool to assess post-IRE ablation changes. Although it is not ideal, its wide availability makes it prudent that we understand its utility in this patient subset. It cannot be overstated that serial imaging over at least 2–6 months must be employed to detect recurrence by comparing with prior studies in conjunction with clinical and serum studies. A single CT image time point is not sufficient to document a recurrence in patients with locally advanced pancreatic cancer given the heterogeneity of the tissue being imaged, the ongoing IRE effects, and the limitations of the modality as described in this paper. Further studies are needed to evaluate a more ideal imaging modality for this unique patient population.

## References

[1] Jemal A., Bray F., Center M. M., Ferlay J., Ward E., Forman D. (2011). Global cancer statistics. *CA—A Cancer Journal for Clinicians*.

[2] Espey D. K., Wu X.-C., Swan J. (2007). Annual report to the nation on the status of cancer, 1975-2004, featuring cancer in American Indians and Alaska Natives. *Cancer*.

[3] Siegel R., Ma J., Zou Z., Jemal A. (2014). Cancer statistics, 2014. *CA: A Cancer Journal for Clinicians*.

[4] Arnoletti J. P., Frolov A., Eloubeidi M. (2011). A phase i study evaluating the role of the anti-epidermal growth factor receptor (EGFR) antibody cetuximab as a radiosensitizer with chemoradiation for locally advanced pancreatic cancer. *Cancer Chemotherapy and Pharmacology*.

[5] Bilimoria K. Y., Bentrem D. J., Merkow R. P. (2007). Application of the pancreatic adenocarcinoma staging system to pancreatic neuroendocrine tumors. *Journal of the American College of Surgeons*.

[6] Varshney S., Sewkani A., Sharma S. (2006). Radiofrequency ablation of unresectable pancreatic carcinoma: feasibility, efficacy and safety. *Journal of the Pancreas*.

[7] Matsui Y., Nakagawa A., Kamiyama Y., Yamamoto K., Kubo N., Nakase Y. (2000). Selective thermocoagulation of unresectable pancreatic cancers by using radiofrequency capacitive heating. *Pancreas*.

[8] Nakagawa A., Kamiyama Y., Matsui Y. (1996). Selective thermocoagulation of unresectable malignant tumors using radiofrequency. *Gan To Kagaku Ryoho*.

[9] Carrafiello G., Ierardi A. M., Fontana F. (2013). Microwave ablation of pancreatic head cancer: safety and efficacy. *Journal of Vascular and Interventional Radiology*.

[10] Carrafiello G., Ierardi A. M., Piacentino F. (2012). Microwave ablation with percutaneous approach for the treatment of pancreatic adenocarcinoma. *CardioVascular and Interventional Radiology*.

[11] Tang Z., Wu Y.-L., Fang H.-Q. (2008). Treatment of unresectable pancreatic carcinoma by radiofrequency ablation with ‘cool-tip needle’: report of 18 cases. *National Medical Journal of China*.

[12] Martin R. C. G., McFarland K., Ellis S., Velanovich V. (2012). Irreversible electroporation therapy in the management of locally advanced pancreatic adenocarcinoma. *Journal of the American College of Surgeons*.

[13] Martin R. C., McFarland K., Ellis S., Velanovich V. (2013). Irreversible electroporation in locally advanced pancreatic cancer: potential improved overall survival. *Annals of Surgical Oncology*.

[14] Narayanan G., Hosein P. J., Arora G. (2012). Percutaneous irreversible electroporation for downstaging and control of unresectable pancreatic adenocarcinoma. *Journal of Vascular and Interventional Radiology*.

[15] Bagla S., Papadouris D. (2012). Percutaneous irreversible electroporation of surgically unresectable pancreatic cancer: a case report. *Journal of Vascular and Interventional Radiology*.

[16] Zhang Y., Guo Y., Ragin A. B. (2010). MR imaging to assess immediate response to irreversible electroporation for targeted ablation of liver tissues: preclinical feasibility studies in a rodent model. *Radiology*.

[17] Appelbaum L., Ben-David E., Sosna J., Nissenbaum Y., Goldberg S. N. (2012). US findings after irreversible electroporation ablation: radiologic-pathologic correlation. *Radiology*.

[18] Martin R. C. G. (2013). Irreversible electroporation of locally advanced pancreatic head adenocarcinoma. *Journal of Gastrointestinal Surgery*.

[19] Bower M., Sherwood L., Li Y., Martin R. (2011). Irreversible electroporation of the pancreas: definitive local therapy without systemic effects. *Journal of Surgical Oncology*.

[20] Charpentier K. P., Wolf F., Noble L., Winn B., Resnick M., Dupuy D. E. (2010). Irreversible electroporation of the pancreas in swine: a pilot study. *HPB*.

[21] Solbiati L., Goldberg S. N., Ierace T. (1997). Hepatic metastases: percutaneous radio-frequency ablation with cooled-tip electrodes. *Radiology*.

[22] Solbiati L., Ierace T., Goldberg S. N. (1997). Percutaneous US-guided radio-frequency tissue ablation of liver metastases: treatment and follow-up in 16 patients. *Radiology*.

[23] Choi H., Loyer E. M., DuBrow R. A. (2001). Radio-frequency ablation of liver tumors: assessment of therapeutic response and complications. *Radiographics*.

[24] Ahmed M., Brace C. L., Lee F. T., Goldberg S. N. (2011). Principles of and advances in percutaneous ablation. *Radiology*.

[25] Delbeke D., Martin W. H. (2005). Update of PET and PET/CT for hepatobiliary and pancreatic malignancies. *HPB*.

